# Association between age-related factors and extubation failure in elderly patients

**DOI:** 10.1371/journal.pone.0207628

**Published:** 2018-11-20

**Authors:** Raveewan Suraseranivong, Orapitchaya Krairit, Pongdhep Theerawit, Yuda Sutherasan

**Affiliations:** 1 Department of Internal Medicine, Division of Geriatric Medicine, Faculty of Medicine, Ramathibodi Hospital, Mahidol University, Bangkok, Thailand; 2 Department of Internal Medicine, Division of Pulmonary and Critical Care Medicine, Faculty of Medicine, Ramathibodi Hospital, Mahidol University, Bangkok, Thailand; Azienda Ospedaliero Universitaria Careggi, ITALY

## Abstract

**Background:**

Elderly patients are being increasingly admitted to the intensive care unit (ICU) for mechanical ventilation. Previous studies demonstrated that 20% to 35% of elderly patients were reintubated within 48 to 72 hours after extubation. Given the age-related physiologic changes and multiple comorbidities in elderly patients, the current conventional parameters for predicting extubation outcomes may not be applicable to this population. This study was performed to identify the association between age-related parameters and extubation failure in elderly patients.

**Methods:**

Intubated elderly patients (age of ≥60 years) admitted to the medical ICU of a university-based hospital from October 2014 to July 2015 were included. Failed extubation was defined as reintubation within 48 hours after the first extubation. The associations of extubation failure with demographic data, vital signs, cognition and anxiety, and ventilator parameters at the time of intubation and extubation were analyzed.

**Results:**

In total, 127 intubated elderly patients were recruited. Extubation failure occurred in 15 patients (11.8%). Patients with failed extubation had a lower body temperature (37.0°C vs. 37.3°C, P < 0.05) but a higher Facial Anxiety Scale (FAS) score than those with successful extubation (3 vs. 2, P < 0.05). Patients with extubation failure had significantly higher levels of blood urea nitrogen (BUN) (39.88 vs. 58.47 g/dL), serum sodium (137.66 vs. 141.47 mmol/L), and serum calcium (9.52 vs. 10.0 g/dL) but a wider anion gap (12.23 vs. 9.97), but no significant differences in respiratory parameters were found between the two groups. Multiple logistic regression revealed no independent factors associated with successful extubation.

**Conclusion:**

This study revealed no strong predictive factors. However, several physiological parameters (lower body temperature and higher FAS scores) and metabolic parameters (BUN, sodium, calcium, and anion gap) were significantly associated with the rate of extubation failure.

## Background

Elderly patients are being increasingly admitted to hospitals, particularly critical care units. The incidence of acute respiratory failure increases exponentially with age. The incidence of extubation failure in the general population ranges from 3% to 19% [1]. The incidence of acute respiratory failure of any cause is more than three times higher in patients aged >65 years than in the younger population [2].

Advanced age is an important factor associated with an increased risk of extubation failure. In previous studies, 20% to 35% of elderly patients were reintubated within 48 to 72 hours after extubation [1, 3]. This number is higher than that in the general population. Additionally, several studies have shown that advanced age is an important risk factor for failed extubation [1, 4–6].

Because of age-related physiologic changes, older adults develop stiffening of the thoracic cage, higher residual volume, weakened diaphragmatic and respiratory muscle strength, decreased sensitivity of the cough center of the brain, and a decline in cardiac function [7]. All of these factors contribute to greater difficulty of extubation and weaning from mechanical ventilation in elderly patients than in the adult general population. In a previous study, the rapid shallow breathing index (RSBI) in elderly patients was >130, while that in patients in the general population was <105 [8]. A few studies have also shown that many classic predictors such as the respiratory frequency–tidal volume ratio, negative inspiratory force, and minute ventilation in the evaluation prior to possible extubation cannot be used to predict the risk of extubation failure in elderly patients [1, 5, 9]. Structural changes in the diaphragmatic muscle and chest wall lead to a decline in the maximal inspiratory pressure, vital capacity, and maximal voluntary ventilation [7]. Therefore, the weaning parameters in elderly patients should be different from those in the general population.

Given the above physiologic changes and previous study findings, we aimed to identify the parameters that can predict successful extubation in elderly patients.

## Methods

Our research has been approved by the Faculty of Medicine, Ramathibodi hospital Institutional Review Board (IRB) and all clinical investigation have been conducted according to the principles expressed in the Declaration of Helsinki. Written Informed consents have been obtained from the participants.

### Study population

We prospectively recruited patients from the medical intensive care unit (ICU) at Ramathibodi Hospital, a university-based hospital in Thailand, from October 2014 and July 2015. The study was approved by the institutional review board of Ramathibodi Hospital, Mahidol University. We included patients aged >60 years who required invasive mechanical ventilation and excluded patients who had undergone tracheostomy, had end-stage disease, died prior to extubation, and were uncooperative.

### Definition of extubation failure

Mechanical ventilation was discontinued and patients were extubated under the direction of the attending pulmonary or critical care physicians in the ICU or intermediate care ward. The patients were closely observed after extubation. If signs of respiratory failure were observed, the patients were reintubated immediately. Extubation failure was defined as reintubation within 48 hours after extubation and restarting of invasive mechanical ventilation [1, 3, 10].

### Data collection and definitions

We recorded demographic and clinical data including age, sex, comorbidities, history of smoking, and duration of admission. The etiologies of respiratory failure were also recorded and categorized as shown in Table 1.

**Table 1:** Etiologies of respiratory failure.

Etiologies	Characteristics
Acute hypoxic respiratory failure (type I)	Any pulmonary pathology associated with impaired gas exchange due to either alveolar-filling pathology or decreased pulmonary blood flow (e.g., pulmonary embolism, cardiac arrest, pneumonia, pulmonary edema)
Hypercapnic respiratory failure (type II)	Any process associated with an excessive respiratory load, impaired neuromuscular function, or decreased ventilatory drive (e.g., obstructive lung disease, respiratory muscle weakness)
Metabolic respiratory failure	Any state of a severe drainage acid-base disorder leading to the need for mechanical ventilation (e.g., diabetic ketoacidosis, lactic acidosis from septic shock)
Respiratory failure related to airway protection	Any condition that requires mechanical ventilation to protect the airway and respiration and is not considered evidence of organ failure

Data during admission were recorded at two time points. Because of the tertiary care level for older adults, our hospital received referred patients who were already intubated. Thus, our first data collection was performed 24 hours after admission if the patient was intubated upon ICU arrival or 24 hours after intubation. The second data set was collected prior to extubation. The following ICU-related data were recorded: vital signs, severity of illness according to the Acute Physiology and Chronic Health Evaluation II (APACHE II) score, duration of ICU stay, mortality outcome, mode of mechanical ventilation, duration of mechanical ventilation, respiratory parameters (PaO_2_/FiO_2_, tidal volume and minute ventilation before extubation, and RSBI), laboratory results (hemoglobin, blood urea nitrogen [BUN], creatinine, sodium, potassium, calcium, magnesium, phosphate, anion gap, albumin, and total protein), steroid treatment, and sedative agents given during the intubation period.

Each patient’s level of consciousness and cognition were determined using the Glasgow coma scale (GCS), a standardized assessment of level of consciousness [11]; the Richmond Agitation-Sedation scale (RASS), another measurement of the level of consciousness in the ICU setting [12]; the Thai version of the Confusion Assessment Method for the ICU (CAM-ICU), which is used to evaluate patients with delirium in the intubated state [13]; and the Faces Anxiety Scale (FAS), an image-based reference for assessing a patient’s stress level [14].

Because the patients were intubated, assessment of their verbal responses was limited. Therefore, we assessed only eye opening and motor response on the GCS in this study, resulting in a maximum score of 10 (E4V_T_M6). The reintubation rate was also determined.

### Statistical analysis

Data were compared between patients who were successfully extubated and patients who underwent failed extubation. Categorical data were compared using the chi-square test. All continuous data were tested for a normal distribution using the Kolmogorov–Smirnov test. Student’s two-tailed t-test was used to compare normally distributed data. The Mann–Whitney U test was used for non-normally distributed variables.

A multiple logistic regression model was used to identify independent factors. All statistical analyses were performed by SPSS v.17 (SPSS Inc., Chicago, IL, USA).

## Results

In total, 155 mechanically ventilated patients were recruited in this study. Twenty-eight patients were excluded due to undergone tracheostomy, the presence of end-stage disease, or death. Thus, 127 patients were eligible for inclusion in our study. Extubation failure was observed in 15 patients (11.8%).

### Baseline characteristics

Comparison of the two groups revealed no differences in the median age, sex, comorbidities, causes of respiratory failure, or history of smoking. Successfully weaned patients had a shorter duration in the ICU (8.5 vs. 16 days, P = 0.01) and a lower mortality rate (12.5% vs. 53.33%, P < 0.01) than patients with extubation failure (Table 2).

**Table 2:** Patient characteristics.

Baseline characteristics	Failure (n = 15)	Success (n = 112)	P
Age, years	78.73 ± 9.33	75.97 ± 9.16	0.276^#^
Sex			
Male	5 (33.3)	65 (58.3)	0.071*
Female	10 (66.7)	47 (42.0)	
Death	8 (53.33)	14 (12.5)	0.000*
ICU duration, days	16 (4–41)	8.5 (1–55)	0.01^+^
Duration of admission, days	24 (4–45)	21 (3–89)	0.411^+^
Mechanical ventilation, days	7 (1–12)	4.5 (1–53)	0.454^+^
**Comorbidities**			
COPD	1 (6.7)	20 (17.9)	0.273*
Other pulmonary disease	1 (6.7)	21 (18.8)	0.246*
Heart disease	9 (40.0)	44 (39.3)	0.958*
Stroke	3 (20.0)	25 (22.3)	0.839*
Other neurologic disease	3 (20.0)	26 (23.2)	0.781*
Dementia	1 (6.7)	19 (17.0)	0.304*
DM	9 (60.0)	50 (44.6)	0.263*
CKD	3 (20.0)	39 (34.8)	0.252*
Liver disease	2 (13.3)	9 (8.0)	0.493*
Malignancy	2 (13.3)	19 (17.0)	0.770*
Other underlying disease	8 (53.3)	84 (75.0)	0.078*
No known underlying disease	0 (0.0)	4 (3.6)	0.457*
Smoking	4 (26.7)	40 (36.7)	0.447*
**Cause of respiratory failure**			
Acute hypoxic respiratory failure	6 (40.0)	52 (46.4)	0.712*
Hypercapnic respiratory failure	1 (6.7)	16 (14.3)	
Metabolic respiratory failure	3 (20.0)	18 (16.1)	
Respiratory failure related to airway protection	5 (33.3)	26 (23.2)	

Data are presented as mean ± standard deviation, n (%), or median (range).

*Chi-square test.

^#^Student’s t-test.

^+^Mann–Whitney U test.

ICU, intensive care unit; COPD, chronic obstructive pulmonary disease; DM, diabetes mellitus; CKD, chronic kidney disease.

### Clinical data on first day of intubation

As shown in Table 3, patients with extubation failure had a lower body temperature than those with successful extubation (37.0°C vs. 37.3°C, P = 0.037), but other vital signs were not significantly different between the two groups. The successfully extubated patients had lower FAS scores than patients with extubation failure (3 vs. 2, P = 0.023), indicating a lower level of anxiety. The median RASS score was not different between the two groups.

**Table 3:** Patient presentation on first day of intubation.

Presentation on first day of intubation	Failure (n = 15)	Success (n = 112)	P
**Vital signs**			
Temperature, ^o^C	37 (34.5–38.3)	37.3 (35.0–40.2)	0.037^+^
Heart rate, beats/min	98.4 ± 21.69	98.01 ± 23.07	0.951^#^
Respiratory rate, breaths/min	24 (16–36)	23.5 (12–40)	0.874^+^
MAP, mmHg	84.95 ± 0.90	82.54 ± 16.45	0.607^#^
GCS score	9 (2–10)	10 (2–10)	0.525^+^
APACHE II score	20.27 ± 10.77	20 ± 7.20	0.898^#^
**Level of consciousness**			
Delirium (CAM-ICU–positive)	11 (73.3)	75 (67.0)	0.620*
RASS score	0 (−5–1)	0 (−5–1)	0.206^+^
FAS score	3 (1–4)	2 (0–4)	0.023^+^
**Ventilator mode**			
PCV	12 (80.0)	98 (87.5)	0.633*
VCV	1 (6.7)	3 (2.7)	
PSV	2 (13.3)	11 (9.8)	
PaO_2_/FiO_2_	262 (48–545)	310 (61–1087)	0.131^+^
**Laboratory results**			
pH	7.42 ± 0.94	7.44 ± 0.09	0.361^#^
Hb, g/dL	10.01 ± 2.05	10.60 ± 2.14	0.319^#^
BUN, g/dL	29 (12–79)	28.5 (3–97)	0.737^+^
Cr, g/dL	1.18 (0.32–4.29)	1.2 (0.33–7.87)	0.545^+^
Na, mmol/L	137.6 ± 6.10	135.49 ± 8.49	0.355^#^
K, mmol/L	3.85 ± 6.39	4.07 ± 0.69	0.263^#^
Ca (g/dL)	9.80 ± 0.92	9.32 ± 0.83	0.048 ^#^
Mg (g/dL)	1.90 ± 0.38	2.00 ± 0.45	0.415^#^
PO_4_ (g/dL)	3.6 (2.5–10.7)	3.3 (0.8–11.6)	0.372^+^
Anion gap	12.81 ± 4.18	12.58 ± 5.76	0.886^#^
Albumin (mg/dL)	27.34 ± 8.74	26.81 ± 6.67	0.787^#^
Total protein (g/dL)	68.78 ± 10.12	63.27 ± 11.02	0.105^#^

Data are presented as mean ± standard deviation, n (%), or median (range).

*Chi-square test.

^#^Student’s t-test.

^+^Mann–Whitney U test.

MAP, mean arterial pressure; GCS, Glasgow coma scale; APACHE II, Acute Physiology and Chronic Health Evaluation II; RASS, Richmond Agitation-Sedation Scale; CAM-ICU, Confusion Assessment Method for the ICU; FAS, Facial Anxiety Scale; PCV, pressure-controlled ventilation; VCV, volume-controlled ventilation; PSV, pressure support ventilation; Hb, hemoglobin; BUN, blood urea nitrogen; Cr, creatinine; Na, sodium; K, potassium; Ca, calcium; Mg, magnesium; PO_4_, phosphate.

Laboratory examination on the first day of intubation showed that the serum calcium level was significantly lower in patients with successful than failed extubation (9.32 ± 0.83 vs. 9.80 ± 0.92, P = 0.048).

### Clinical data on day of extubation

On the day of extubation, no significant differences were found in the ventilation parameters, including the RSBI. With respect to the patients’ levels of cognition and delirium, there were no differences in the RASS score and CAM-ICU results between the two groups as shown in Table 4.

**Table 4:** Patient presentation on day of extubation.

Presentation on day of extubation	Failure (n = 15)	Success (n = 112)	P
**Type of weaning process**			
SBT	7 (46.7)	61 (54.5)	0.827*
PSV	4 (26.7)	23 (20.5)	
External CPAP	4 (26.7)	25 (22.3)	
Self-extubation	0 (0.0)	3 (2.7)	
**Respiratory parameters**			
NIV	9 (60.0)	56 (50.0)	0.476*
Tidal volume	391.67 ± 151.87	394.07 ± 134.06	0.949^#^
Minute ventilation	8.85 ± 3.85	8.69 ± 3.74	0.878^#^
RSBI	61.6 (13–209)	56 (7–152)	0.368^+^
Vital capacity	850 ± 264.58	876.7 ± 399.58	0.899^#^
**Level of consciousness**			
FAS score	3 (1–4)	2 (1–5)	0.693^+^
Delirium (CAM-ICU)	9 (60.0)	63 (56.3)	0.783*
RASS score	0 (−5–1)	0 (−5–1)	0.753^+^
**Laboratory results**			
Hb, g/dL	9.58 ± 1.34	10.16 ± 1.48	0.152^#^
BUN, g/dL	58.47 ± 35.17	39.88 ± 25.48	0.013 ^#^
Cr, g/dL	1.94 (0.53–6.89)	1.29 (0.33–11.39)	0.560^+^
Na, mmol/L	141.47 ± 7.59	137.66 ± 5.94	0.026^#^
K, mmol/L	4.026 ± 0.65	3.988 ± 0.474	0.784^#^
Ca, g/dL	10.07 ± 0.93	9.52 ± 0.62	0.012^#^
Albumin, mg/dL	23.06 ± 5.52	23.08 ± 5.97	0.990^#^
Mg, g/dL	2.02 ± 0.25	2.05 ± 0.35	0.731^#^
PO_4_, g/dL	3.98 ± 0.78	3.65 ± 1.35	0.459^#^
Anion gap	12.23 ± 2.84	9.97 ± 3.79	0.028^#^

Data are presented as mean ± standard deviation, n (%), or median (range).

*Chi-square test.

^#^Student’s t-test.

^+^Mann–Whitney U test.

SBT, spontaneous breathing trial; PSV, pressure support ventilation; CPAP, continuous positive airway pressure; NIV, noninvasive ventilation; RSBI, rapid shallow breathing index; RASS, Richmond Agitation-Sedation Scale; CAM-ICU, Confusion Assessment Method for the ICU; FAS, Facial Anxiety Scale; Hb, hemoglobin; BUN, blood urea nitrogen; Cr, creatinine; Na, sodium; K, potassium; Ca, calcium; Mg, magnesium; PO_4_, phosphate.

Laboratory tests showed that the levels of BUN, serum sodium, and serum calcium were lower in the patients with successful extubation (39.88 ± 25.48 vs. 58.47 ± 35.17, P = 0.013; 137.66 ± 5.94 vs. 141.47 ± 7.59, P = 0.026; and 9.52 ± 0.62 vs. 10.0 ± 0.93, P = 0.012, respectively) and that the anion gap was wider in the patients with failed extubation (12.23 ± 2.84 vs. 9.97 ± 3.79, P = 0.028).

### Medication

As shown in Table 5, steroids and sedative drugs were used more frequently in patients with failed than successful extubation, but without a statistically significant difference.

**Table 5:** Medication using during intubation.

Medication	Failure (n = 15)	Success (n = 112)	P
Steroids	8 (53.3)	40 (35.7)	0.186*
Sedative drugs	10 (66.7)	52 (46.4)	0.141*

Data are presented as n (%).

*Chi-square test.

Multiple logistic regression was performed for comparison among all parameters. The results showed that there were no independent factors associated with successful extubation.

## Discussion

In the present prospective cohort study, 11.8% of patients had extubation failure. This finding is consistent with previous studies in which the incidence of extubation failure varied from 6% to 47% [15]. Patients who were reintubated within 48 to 72 hours had higher mortality outcomes and longer ICU stays. The increased mortality rate after extubation or weaning failure may reflect the serious illness of the study population [16].

### Risk factors for extubation failure

The results of this study demonstrated that age, sex, comorbidities, and causes of respiratory failure were not associated with successful extubation. This contradicts the findings of several other studies showing that older age, underlying chronic respiratory disease, and neurological impairment were associated with a higher risk of extubation failure [1, 9, 15]. Because almost all patients in both groups were >65 years of age and had more than one comorbidity, both groups were already considered to be at high risk for extubation failure. Additionally, the severity of illness and baseline physical function varied among the patients. The comparison between successful and failed extubation showed no differences because of these confounding factors. Therefore, future studies should include baseline physical function and indexes of comorbidities, such as the Charlson comorbidity index, as baseline clinical parameters for predicting extubation failure [17].

The parameters used to predict successful extubation vary among previous studies. In the present study, we selected the parameters deemed critical and possibly causative of extubation failure, such as anxiety, delirium, the RSBI, and blood chemistry results. The parameters in this study were compared on the first day of intubation and on the day of extubation.

This study demonstrated that many parameters were associated with successful extubation. Nevertheless, the multiple logistic regression analysis revealed no independent factors associated with successful extubation. Many variables are likely to be closely associated with one another, and application of a single parameter alone can be misleading. The general status of the patient, including vital signs, cognition, and laboratory parameters, should all be taken into account for successful weaning.

Theoretically, thermoregulatory functions deteriorate with advancing age because of the decreasing protective function of all body processes. These changes directly impact the ability of elderly patients to maintain thermal hemostasis [18]. A lower body temperature in the ICU reflects more serious illness and less physiologic reserve than does a normal body temperature. This can explain why patients with a lower body temperature had a higher rate of failed extubation in the present study.

Mood and cognition have been discussed as factors associated with successful extubation. Our study showed that anxiety had an impact on successful extubation. Anxiety is hypothesized to have systemic effects throughout the body. Anxiety is associated with hyperactivity of peripheral adrenergic systems, by which it increases the circulating concentrations of plasma ACTH and affects inflammation and immune dysregulation [19,20]. These effects lead to decreased physiologic reserve in elderly patients and a higher risk of reintubation in extubated patients. Although delirium is frequent in the ICU, our study showed that this variable was not significantly associated with extubation failure, consistent with a study by Thille et al [9].

The RSBI is one of the most commonly used indices for prediction of weaning [21]. Several studies have shown that a low respiratory frequency–tidal volume ratio is associated with a high likelihood of successful extubation. In the present study, consistent with the findings reported by El Solh et al. [1], the RSBI was not significantly different between patients with successful and failed extubation. Elderly patients undergo age-related changes that increase the residual volume and decrease vital capacity, resulting in an increased respiratory rate and decreased tidal volume [18]. For this reason, the RSBI should not be used in the elderly population. However, Krieger et al. [8] demonstrated that an RSBI of >130 predicted a high risk of extubation failure. Notably, the RSBI in their study was a serial parameter measured from the start of spontaneous breathing until 5 hours prior to extubation. The results of their study more accurately reflect the endurance and physiologic reserve of elderly patients than measurement at one time point, as in our study.

In the present study, lower levels of BUN, serum sodium, and serum calcium were associated with successful extubation. The levels of BUN and serum sodium reflect the retention of waste products in the systemic circulation and imbalance of total body water. Serum calcium is a necessary electrolyte for maintenance of respiratory function. The function of airway smooth muscle, mast cells, mucous glands, and the vagus nerve is dependent upon calcium ions [22]. Therefore, an abnormal serum calcium level is associated with extubation failure. The anion gap was also associated with extubation failure in our study; this is consistent with the findings of Saugel et al. [23]. These results suggest that physicians need to maintain adequate fluid hydration and electrolyte balance prior to extubating patients. In contrast, serum albumin was not associated with extubation, which is differs from the results of another previous study [24]. Serum albumin is a negative acute-phase reactant protein, and its synthesis usually decreases in response to inflammation. Our study population comprised elderly patients with critical illness; thus, the level of serum albumin within the intubation period was expected to be lower than normal. This caused the similarity of the serum albumin level between patients with successful and failed extubation.

### Limitations

This study has several limitations. First, it was conducted in a single center, and almost the entire population was native; therefore, the findings may not be generalizable to all countries in terms of the demographic data and primary reasons for intubation. Second, there were no definite guidelines for ventilator weaning and decisions regarding extubation, likely resulting in some information or confounding bias in the methods of this study. Third, the extubation failure rate in this study was lower than that in previous studies, and the number of patients with extubation failure was not large enough for accurate assessment of predictive factors [1, 3]. Third, multiple logistic regression analysis revealed no independent factors affecting successful extubation, possibly because the variables were closely associated with one another. The general status of the patient, including vital signs, cognition, and laboratory parameters, should all be taken into account for successful weaning. Finally, all parameters used to predict successful extubation were static parameters. Measurement of variables at one time point does not represent the course of the ventilation and extubation process. Therefore, more frequent monitoring of serial parameters that change during the period of intubation and extubation should be performed to identify the parameters that are significantly associated with successful extubation.

## Conclusion

Although no strong predictive factors were found in this study, several physiological parameters (lower body temperature and higher FAS scores) and metabolic parameters (BUN, sodium, calcium, and anion gap) were significantly associated with the rate of extubation failure.

## Supporting information

S1 File This is dataset file. (XLS)
